# Prognostic significance of ground-glass areas within tumours in non-small-cell lung cancer

**DOI:** 10.1093/ejcts/ezae158

**Published:** 2024-04-10

**Authors:** Hiroyuki Sakurai, Yasushi Goto, Kiyotaka Yoh, Kazuya Takamochi, Takehiro Shukuya, Tomoyuki Hishida, Masahiro Tsuboi, Koichi Yoshida, Yasuhisa Ohde, Sakae Okumura, Masataka Taguri, Hideo Kunitoh

**Affiliations:** Division of Respiratory Surgery, Nihon University School of Medicine, Tokyo, Japan; Department of Thoracic Oncology, National Cancer Center Hospital, Tokyo, Japan; Department of Thoracic Oncology, National Cancer Center Hospital East, Kashiwa, Japan; Department of General Thoracic Surgery, Graduate School of Medicine, Juntendo University, Tokyo, Japan; Department of Respiratory Medicine, Graduate School of Medicine, Juntendo University, Tokyo, Japan; Division of Thoracic Surgery, Department of Surgery, Keio University School of Medicine, Tokyo, Japan; Division of Thoracic Surgery, National Cancer Center Hospital East, Kashiwa, Japan; Department of Thoracic Surgery, Tokyo Medical University School of Medicine, Tokyo, Japan; Division of Thoracic Surgery, Shizuoka Cancer Center, Shizuoka, Japan; Department of Thoracic Surgical Oncology, The Cancer Institute Hospital of JFCR, Tokyo, Japan; Department of Data Science, Tokyo Medical University School of Medicine, Tokyo, Japan; Department of Medical Oncology, Japanese Red Cross Medical Center, Tokyo, Japan

**Keywords:** Non-small-cell lung cancer, Lung cancer surgery, T factor, Ground-glass area

## Abstract

**OBJECTIVES:**

To validate or refute the hypothesis that non-small-cell lung cancers (NSCLC) with ground-glass areas (GGA+) within the tumour on high-resolution computed tomography are associated with a more favourable prognosis than those without GGA (GGA−).

**METHODS:**

We analysed data from a multicentre observational cohort study in Japan including 5005 patients with completely resected pathological stage I NSCLC, who were excluded from the Japan Clinical Oncology Group (JCOG) 0707 trial on oral adjuvant treatment during the enrolment period. The patients’ medical and pathological records were assessed retrospectively by physicians and re-staged according to the 8th tumour, node, metastasis edition.

**RESULTS:**

Of the 5005 patients, 2388 (48%) were ineligible for the JCOG0707 trial and 2617 (52%) were eligible but were not enrolled. A total of 958 patients (19.1%) died. Patients with GGA+ NSCLC and pathological invasion ≤3 cm showed significantly better overall survival than others. In patients with tumours with an invasive portion ≤4 cm, GGA+ was associated with better survival. The prognoses of patients with GGA+ T2a and GGA− T1c tumours were similar (5-year overall survival: 84.6% vs 83.1%, respectively). The survival with T2b or more tumours appeared unaffected by GGA, and GGA was not prognostic in these larger tumours.

**CONCLUSIONS:**

Patients with GGA+ NSCLC on high-resolution computed tomography and ≤4 cm invasion size may have a better prognosis than patients with solid GGA− tumours of the same T-stage. However, the presence or absence of radiological GGA has little impact on the prognosis of patients with NSCLC with greater (>4 cm) pathological invasion.

**Figure:**
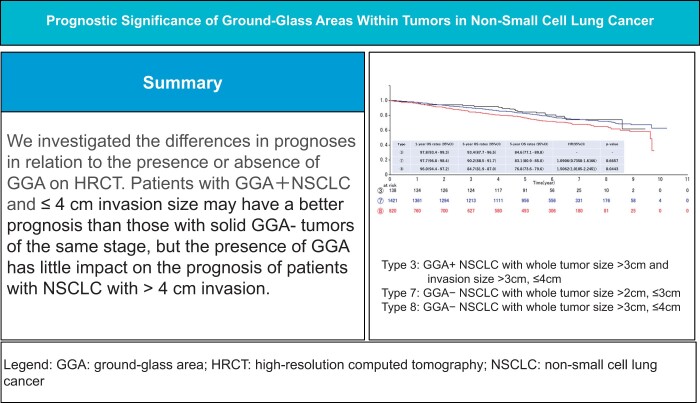

## INTRODUCTION

The International Association for the Study of Lung Cancer published the 8th edition of the International Union Against Cancer tumour, node, metastasis (TNM) classification of lung cancer in 2017 [1]. One of the major changes in this TNM classification was the pathological categorization of T factor by invasive size, rather than maximum whole-tumour size, excluding portions of lepidic tumour growth [1, 2]. This corresponds to clinical T factor defined by the size of the solid component, i.e. excluding ground-glass areas (GGAs) within the tumour on high-resolution computed tomography (HRCT) [3, 4].

The presence of GGA within the tumour has repeatedly been correlated with a better prognosis in patients with non-small-cell lung cancer (NSCLC) [5–11], and stage IA NSCLC with GGA (GGA+) within the tumour on HRCT had a better prognosis than NSCLC with solid components without GGA (GGA−) [6–9]. Indeed, some researchers have advocated adding information on the presence or absence of GGA within the tumour to the clinical T descriptor in future editions of the TNM classification for lung cancer [8, 12]. However, the correlation between GGA within the tumour and prognosis in patients with larger, T2 and more advanced T-stage lung cancers remains controversial.

In the present study, we carried out a sub-analysis of data from a multicentre observational cohort study (CSPOR LC-03) [13] of patients who were excluded from the Japan Clinical Oncology Group (JCOG) 0707 study [14]. Since the patients were excluded from a clinical trial and treated on daily practice, they reflect the real-world outcome of such patients. As were previously reported [13, 15, 16], CSPOR LC-03 study population is heterogenous, with approximately half are trial-eligible ‘fit’ patients and another half trial-ineligible poor-risk ones, making it suitable to clarify what is actually going in daily practice. The study aimed to elucidate the differences in clinicopathologic characteristics and prognoses in relation to the presence or absence of GGA on HRCT or pathological non-invasive areas (lepidic growth component), based on the 8th TNM classification for resected node-negative NSCLC, using a large cohort of real-world data in patients excluded from clinical trial. Our study purpose was to validate or refute the hypothesis that presence of GGA is, in fact, a favourable prognostic factor, even after matched for pathological invasive tumour size.

## PATIENTS AND METHODS

### Patient registry

This was an observational multicentre cohort study conducted in Japan. Patients with completely resected pathological stage I (T1 > 2 cm and T2 in 6th TNM edition) NSCLC confirmed by lobectomy or larger resection with mediastinohilar lymph node dissection (i.e. target population of the JCOG0707 trial [14] but excluded from that trial during that study’s enrolment period) were eligible to participate in this study. The eligibility criteria of JCOG0707 is given in Supplementary Material, Table S1 [14]. The enrolment period of the JCOG0707 trial was November 2008 to December 2013. Researchers from institutions participating in the JCOG0707 trial recorded data from the patients’ medical records, as described previously [13, 15, 16]. Of the 48 institutions participating in JCOG0707, 34 participated in the current observational study. The collected data included the following clinicopathologic and prognostic items: sex, age, presence or absence of GGA within the tumour on HRCT (GGA+ and GGA−, respectively), reason for exclusion from JCOG0707 trial, pathological T factor, mode of surgical procedure, mode of lymph node dissection, tumour diameter, histology, pathological invasion size within the tumour, PL (the pathological extent of pleural invasion defined by the TNM classification), survival time, recurrence and cause of death.

All patients were re-staged according to the recent 8th edition of the International Union Against Cancer TNM Classification of Malignant Tumors [1] (published in 2017) regarding T factor, but pathological invasion size was only measured in GGA+ NSCLCs that were >3 cm in whole-tumour size, as GGA+ NSCLCs with whole-tumour size of ≤3 cm should always have a solid-component size of 3 cm or less. Tumour histology was described according to the 4th edition of the World Health Organization (WHO) classification published in 1999, where pathological invasion was defined when the tumour cells were arranged in acinar/papillotubular structures or solid nests in a fibroblastic stroma, often accompanied by collagenization, and when the alveolar structures were no longer discernible. These pathological findings were diagnosed by expert pathologists at each institute. For GGA−, solid tumours, whole-tumour size was considered to be equal to the invasion size, as shown previously [3, 4]. T factor was thus defined based on solid-component size in radiological GGA− tumours, and pathological invasion size in radiological GGA+ tumours with >3 cm whole-tumour size.

GGA was defined as an area of slight, homogenous increase in density that did not obscure the underlying vascular markings on HRCT. Additionally, PL1 tumours not exposed to the visceral pleural surface were not regarded as a T descriptor, even if the tumour invaded beyond the elastic layer of the visceral pleura but did not extend to the pleural surface of the lung, because T category was registered based on the 6th TNM classification in our study. T category was thus assigned based on pathological invasion size, clinical solid-component size or PL2 pathological pleural invasion extending to the visceral pleural surface.

We categorized the tumour type based on the whole-tumour size, the presence or absence of GGA on HRCT and the pathological invasion size (Supplementary Material, Table S2), with types 1–6 considered as the GGA+ group and types 7–11 considered as the GGA− group.

Tumour histology was described according to the 4th edition of the WHO classification (published in 2015) [17]. Mediastinohilar lymph node dissection was performed according to the ‘systematic’ or ‘lobe-specific’ dissection modes [18, 19].

### Ethical statement

This study was conducted according to the Declaration of Helsinki and approved by the institutional review board of each participating institute, as well as the ethics committee of the Public Health Research Foundation. This was an observational study, and the need for signed informed consent was waived according to the Japanese Ethical Guidelines on Scientific Research [20]. The trial was registered with the UMIN Clinical Trials Registry (UMIN000015732).

### Statistical analysis

Differences in categorical and continuous variables were evaluated by χ^2^ tests and one-way analysis of variance, respectively. Survival time was defined as the time between the date of surgery and death with patients without event being censored at the last follow-up date. Survival curves were estimated by the Kaplan–Meier method, and differences in survival were assessed by the log-rank test. Overall survival (OS) was defined as the time between surgery and death from any cause. We used the reverse Kaplan–Meier survival curve [21], which is constructed by reversing ‘censor’ and ‘event (death)’, to compute the median follow-up period. Multivariable Cox’s proportional hazards analysis was used to adjust the significance of factors essential for prognoses according to clinicopathological background for NSCLC, including sex, age, mode of lymph node dissection, histology (adenocarcinoma versus others), tumour size, p-T status (T1 versus others) and adjuvant therapy (tegafur/uracil and others). Significance was defined as a *P* value <0.05. All data analyses were performed with SAS version 9.4 (SAS Institute, Cary, NC).

## RESULTS

### Patients

**Figure 1:**
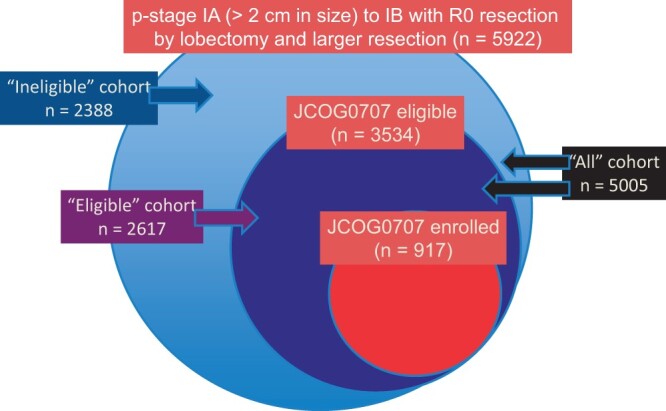
Composition of patients in the present study and JCOG0707 trial. ‘Eligible’ cohort included patients who met the criteria for JCOG0707, but were not enrolled in the study. ‘Ineligible’ cohort included patients who were ineligible for JCOG0707 trial. JCOG: Japan Clinical Oncology Group.

Of the 48 institutions that participated in the JCOG0707 trial, 34 institutions, which enrolled 917 (95%) of the 963 patients registered in the JCOG0707 trial, participated in the current study. During the JCOG0707 trial accrual period, a total of 5922 patients underwent surgical completely resection for pathological stage I (T1 > 2 cm and T2 in 6th TNM edition) NSCLC. Excluding the 917 patients who participated in the JCOG0707 trial, 5005 patients (‘All’ cohort) were enrolled in this observational study. The patient recruitment strategy is shown in Fig. 1. Overall, 85% of patients (5005 of 5922 patients) were excluded from the JCOG0707 trial, including 2388 (48%) who were ineligible for the trial (‘Ineligible’ cohort) and 2617 (52%) who were eligible but were not enrolled for various reasons, including patient refusal or temporary suspension of accrual (‘Eligible’ cohort) [16]. The 5005 patients included 2916 males (58%) and 2089 females (42%), aged 20–93 years (median, 69 years). Approximately one-third of the patients (1667; 33%) received adjuvant therapy. Most of them (1549 or 93% of those with adjuvant therapy) were treated with standard tegafur/uracil. The impact of adjuvant tegafur/uracil on patient outcome has been reported previously [13]. Among the patients who received ‘other’ adjuvant chemotherapy, the majority received platinum-base, and very few (3 patients; 0.1%) received epidermal growth factor receptor (EGFR)-tyrosine kinase inhibitor.

### Clinicopathological findings

The clinicopathologic characteristics of the patients in this study and those accrued to the JCOG0707 trial (the ‘JCOG0707’ cohort) have been reported previously [14, 16]. The characteristics of the ‘Eligible’ and ‘Ineligible’ cohorts are re-summarized in Table 1. The ‘All’ cohort comprised 2115 patients (42.3%) of the GGA+ group and 2864 patients (57.2%) of the GGA− group. EGFR mutation status was investigated in 2172 (44%) of the patients, with 831 (17%) mutation positive, and 1341 (27%) wild type. EGFR mutation was observed in 488 (53.5%) of the 906 GGA+ patients, 335 (27.1%) of the 1238 GGA− patients and in 8 patients without information on radiological GGA.

**Table 1:** Clinicopathological features of patients

Characteristic	‘All’ cohort	‘Eligible’ cohort	‘Ineligible’ cohort
	(*n* = 5005)	(*n* = 2617)	(*n* = 2388)
Sex, *n* (%)			
Male	2916 (58.3)	1402 (53.6)	1514 (63.4)
Female	2089 (41.7)	1215 (46.4)	874 (36.6)
Age (years), *n* (%)			
<70	2517 (50.3)	1556 (59.5)	961 (40.2)
70–79	2098 (41.9)	1020 (39.0)	1078 (45.2)
>80	390 (7.8)	41 (1.5)	349 (14.6)
Radiologic findings within the tumour on HRCT, *n* (%)			
GGA+	2115 (42.3)	1177 (45.0)	938 (39.3)
GGA−	2864 (57.2)	1422 (54.3)	1442 (60.4)
Missing data	26 (0.5)	18 (0.7)	8 (0.3)
Mode of operation, *n* (%)			
Pneumonectomy	23 (0.5)	10 (0.4)	13 (0.5)
Bilobectomy	75 (1.5)	28 (1.1)	47 (2.0)
Lobectomy	4907 (98.0)	2579 (98.5)	2328 (97.5)
Lymph node dissection, *n* (%)			
Systematic	2276 (45.5)	1235 (47.2)	1041 (43.6)
Lobe-specific	2729 (54.5)	1382 (52.8)	1347 (56.4)
Histology, *n* (%)			
Adenocarcinoma	3761 (75.1)	2084 (79.6)	1677 (70.2)
Squamous carcinoma	948 (18.9)	403 (15.4)	545 (22.8)
Other	296 (6.0)	130 (5.0)	166 (7.0)
Pathological stage (6th TNM), *n* (%)			
IA (>2 cm)	2536 (50.7)	1390 (53.1)	1146 (48.0)
IB (T2)	2469 (49.3)	1227 (46.9)	1242 (52.0)
Adjuvant therapy, *n* (%)			
None	3338 (66.6)	1484 (56.7)	1854 (77.6)
UFT	1549 (31.0)	1061 (40.5)	488 (20.5)
Other	118 (2.4)	72 (2.8)	46 (1.9)

GGA: ground-glass area; HRCT: high-resolution computed tomography; TNM: tumour, node, metastasis; UFT: tegafur/uracil.

### Prognosis

The median follow-up period was 6.0 years. A total of 958 patients (19.1%) died. The 5-year OS rates for the ‘Eligible’, ‘Ineligible’ and ‘JCOG0707’ cohorts were 89.3%, 78.7% and 89.5%, respectively (Supplementary Material, Fig. S1). ‘Ineligible’ patients had significantly poorer OS than patients in the ‘Eligible’ and ‘JCOG0707’ cohorts (*P *<* *0.001), while the survival curves were similar in the latter 2 cohorts, implying that ‘study partition’ itself had no impact on patient outcome.

The OS curves according to the mode of lymph node dissection for ‘All’ patients are shown in Supplementary Material, Fig. S2. The survival curves between patients with systematic and selective lymph node dissection were not overall statistically significantly different with a *P*-value of 0.644 and survival rates at time 5 years of 84.1% and 84.5%.

**Figure 2:**
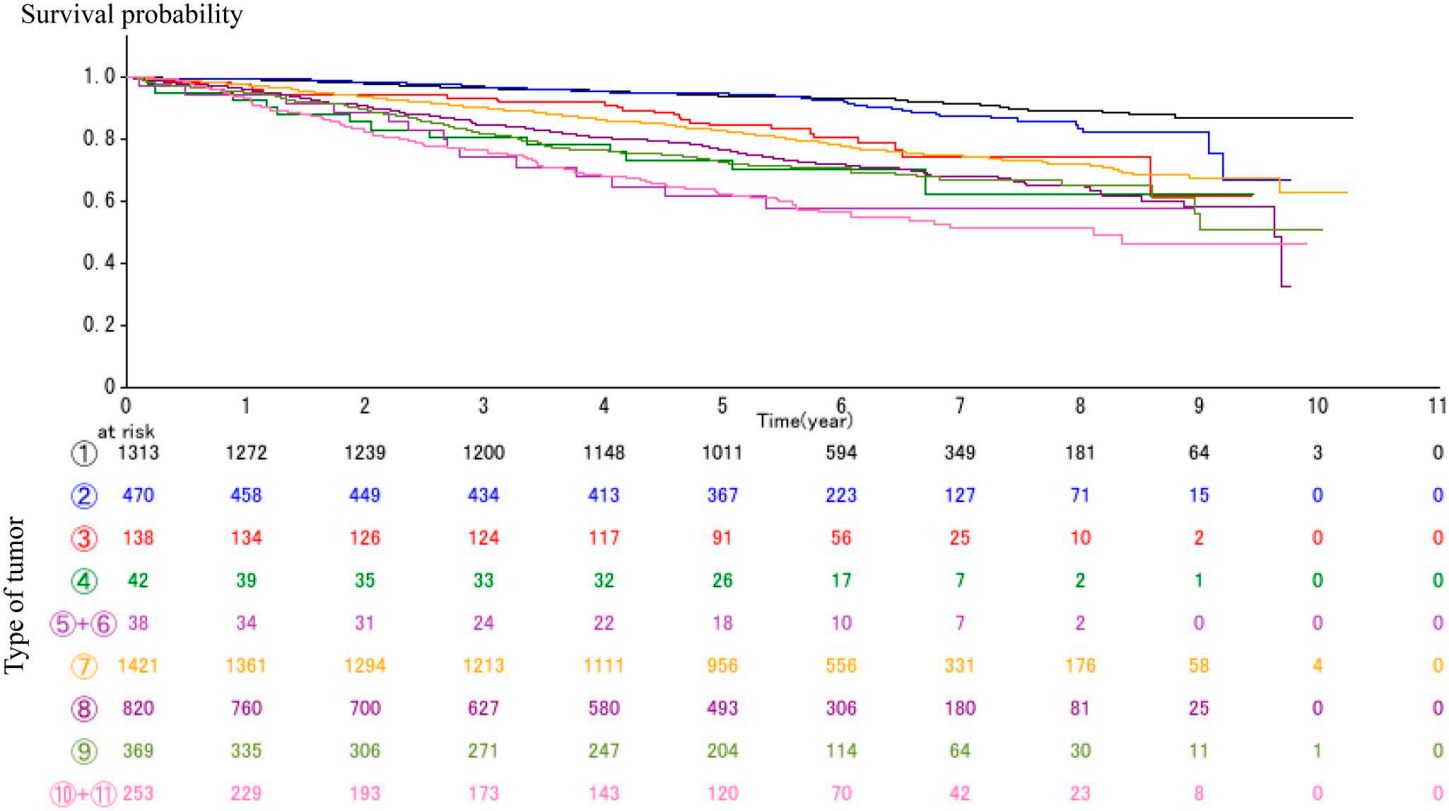
Overall survival curves according to tumour type for the ‘All’ cohort. The 5-year survival rates were 94.1% for type 1, 94.9% for type 2, 84.6% for type 3, 73.4% for type 4, 61.6% for types 5 and 6, 83.1% for type 7, 76.8% for type 8, 72.6% for type 9 and 62.3% for types 10 and 11, respectively.

The OS curves according to tumour type for the ‘All’ cohort are shown in Fig. 2 and the hazard ratios (HRs) of OS are shown in Table 2. The survival curves of patients with types 5 and 6 tumours (GGA+, and >5 cm invasive portion) and patients with types 10 and 11 solid tumours (GGA− and >5 cm invasive portion) were analysed together as ‘large’ tumours, due to the relatively small numbers of patients.

**Table 2:** Multivariable analysis of overall survival according to tumour type in ‘All’ cohort

Tumour type	*n*	5-YSR (%)	Univariable analysis				Multivariable analysis			
			HR	95% CI		*P*-value	HR	95% CI		*P*-value
1	1313	94.1	1.0000				1.0000			
2	470	94.9	1.3150	0.9306	1.8583	0.1206	0.7571	0.5040	1.1373	0.1801
3	138	84.6	2.9120	1.9128	4.4332	<0.0001	1.7109	1.0620	2.7561	0.0273
4	42	73.4	4.9571	2.7188	9.0380	<0.0001	2.5223	1.3637	4.6651	0.0032
5 + 6	38	61.6	6.8953	3.8636	12.3062	<0.0001	3.3450	1.7772	6.2960	0.0002
7	1421	83.1	3.1751	2.5260	3.9821	<0.0001	2.2993	1.8056	2.9280	<0.0001
8	820	76.8	4.3747	3.4494	5.5482	<0.0001	2.0259	1.4729	2.7863	<0.0001
9	369	72.6	4.8055	3.6372	6.3491	<0.0001	2.1154	1.4883	3.0066	<0.0001
10 + 11	253	62.3	7.3604	5.5529	9.7562	<0.0001	2.9171	2.0362	4.1791	<0.0001

Cox proportional hazards model (*n* = 5005).

CI: confidence interval; HR: hazard ratio; *n*: number of patients; YSR: year survival rate.

The 5-year OS rates for the ‘All’ cohort were 94.1% for type 1, 94.9% for type 2, 84.6% for type 3, 73.4% for type 4, 61.6% for types 5 and 6, 83.1% for type 7, 76.8% for type 8, 72.6% for type 9 and 62.3% for types 10 and 11, respectively (Fig. 2, Table 2). Patients with GGA+ lung cancer with invasion size ≤3 cm (types 1 and 2) had significantly better OS than others, with the 5-year OS rate exceeding 94%.

We also analysed the OS according to tumour type for the ‘Eligible’ cohort alone, because the ‘Ineligible’ cohort had many non-lung cancer deaths, which obscures the prognostic impact of the tumours, as previously reported [15].

The 5-year OS rates for the ‘Eligible’ cohort were 96.3% for type 1, 96.0% for type 2, 90.3% for type 3, 91.1% for type 4, 69.6% for types 5 and 6, 88.5% for type 7, 83.6% for type 8, 80.6% for type 9 and 65.3% for types 10 and 11, respectively. These showed the same tendency as those of the ‘All’ cohort.

**Figure 3:**
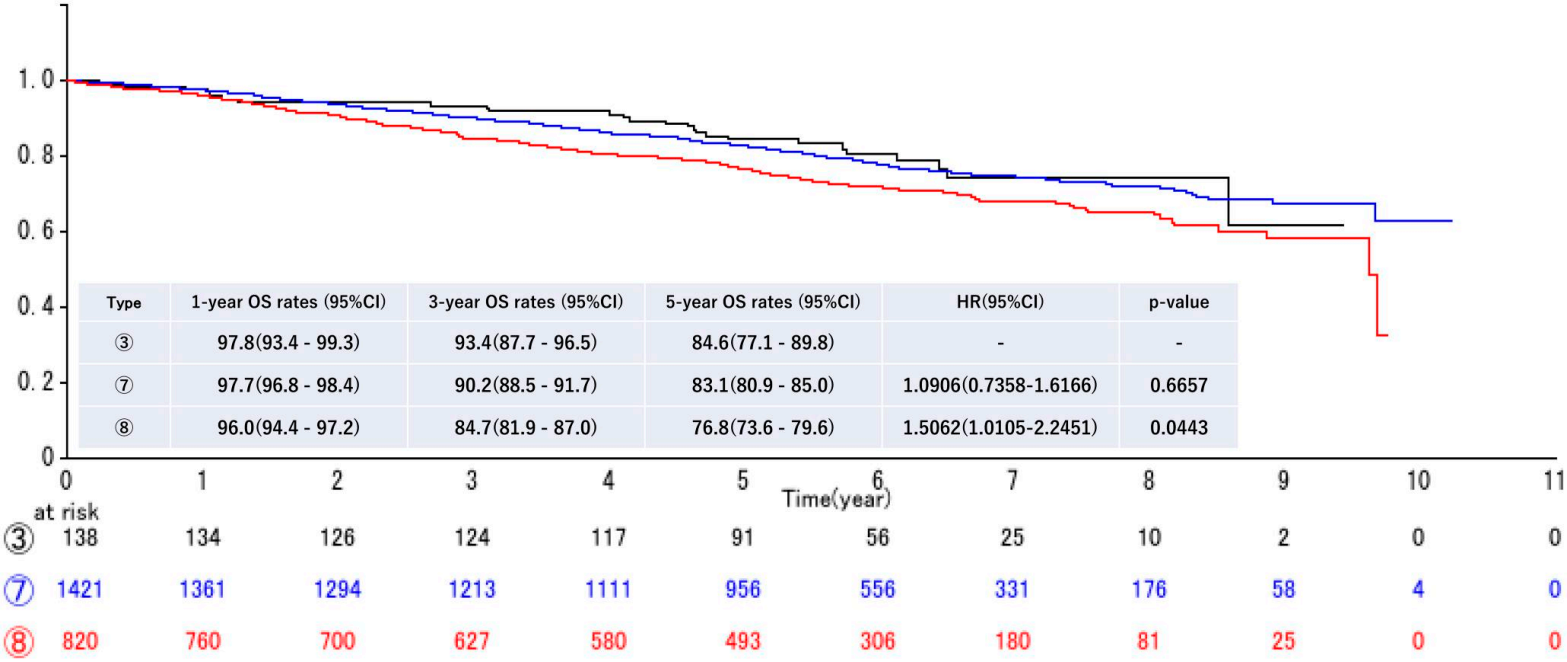
Overall survival curves according to tumour types 3, 7 and 8 for the ‘All’ cohort. The 5-year survival rates were 84.6% for type 3, 83.1% for type 7 and, 76.8% for type 8, respectively. Type 3 (GGA+ T2a) tumour had significantly better prognosis than type 8 (GGA− T2a) tumour (hazard ratio 1.5062, *P *=* *0.0443). CI: confidence interval; HR: hazard ratio; OS: overall survival.

**Figure 4:**
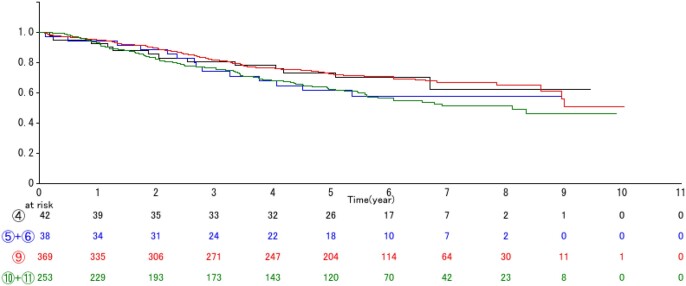
Overall survival curves according to tumour type 4, types 5 + 6, type 9 and, types 10 + 11 for the ‘All’ cohort. The 5-year survival rates were 73.4% for type 4 vs 72.6% for type 9 (hazard ratio 0.9594, *P *=* *0.8895), and 61.6% for types 5 + 6 vs 62.3% types 10 + 11 (hazard ratio 1.0840, *P *=* *0.7786).

In the ‘All’ cohort, as shown in Fig. 3, in patients with tumours with an invasive portion ≤4 cm, GAA+ was associated with better survival. The prognosis of GGA+ T2a tumours (type 3) was comparable to that of GGA− T1c tumours (type 7), and significantly better than that of T-stage-matched type 8 GGA− T2a tumours. The 5-year OS rates were: 84.6% for type 3, 83.1% for type 7 and 76.8% for type 8 (type 3 versus type 8: HR 1.5062, *P *=* *0.0443). However, for T2b or larger tumours, there was no significant difference in OS for NSCLCs of the same T factor (types 4 vs 9: HR 0.9594, *P *=* *0.8895, and types 5 and 6 vs 10 and 11: HR 1.0840, *P *=* *0.7786), regardless of the presence (types 4, 5 and 6) or absence (types 9, 10 and 11) of GGA within the tumour (Fig. 4).

As was previously reported [15], OS was significantly better for EGFR-mutant patients by univariable, but not by multivariable analysis (HR 0.834, *P* = 0.1123), probably reflecting patient backgrounds, such as sex and smoking habits.

## DISCUSSION

As is often the case with prospective clinical trials, only a selected population of candidate patients were enrolled in the JCOG0707 adjuvant chemotherapy trial for early-stage NSCLC. On the other hand, those NOT enrolled in it make a ‘real-world’ cohort, consisting of heterogenous patients. To elucidate the similarities and differences of the trial population and non-trial one, we first analysed the patient outcomes in the heterogenous ‘All’ cohort, then validated the findings in more homogenous and likely to be more cancer-specific ‘Eligible’ cohort. The current observational study reproduced the survival outcome of JCOG0707, especially in the ‘Eligible’ cohort. These data thus provide valuable real-world information, both on prospective trial-eligible and ineligible population, on the outcomes and treatment effectiveness in these patients.

The current 8th edition (and the upcoming 9th edition as well) of the TNM classification of lung cancer categorizes clinical and pathological T factors based on measurement of the invasive component alone. As in preoperative radiologic findings, the solid part of the tumour on HRCT is regarded as the invasive component, whereas GGA within the tumour corresponds to non-invasive areas, where the tumour cells grow along the surface of alveolar walls. In the present study, invasion size was measured pathologically in tumours with GGA (GGA group or GGA+), whereas solid areas on HRCT were considered to indicate the invasion size in tumours without GGA (solid group or GGA−). The analytical methods were based on, and consistent with, previous reports [3, 4] showing that clinical T-stage reflected pathological T-stage, especially in GGA− solid tumours.

Although the present TNM classification decides the T factor based on the size of the solid area, regardless of the presence or absence of GGA within the tumour on HRCT, several recent studies [7–10, 22–24] have reported that radiologic solid tumours (GGA− NCSLC) exhibited more malignant behaviour and had a poorer prognosis compared with GGA+ part-solid tumours, especially for T1-stage IA tumours. They accordingly advocated that the oncologic and prognostic outcomes should be discussed separately for lung cancer patients with and without GGA within the tumour. In addition, previous reports showed that the 5-year survival of patients with T1N0M0 (stage IA) lung cancer with GGA on HRCT was similar, regardless of the size of the solid component [8, 11]. The current study also showed that survival differed between T1-stage IA tumours with and without GGA, even if the T factor, defined by invasion size within the tumour, was classified in the same category. GGA− solid T1 tumours (type 7) showed a significantly poorer prognosis than GGA+ T1 tumours, with no survival difference among GGA+ T1 tumours (types 1 and 2) regardless of the whole-tumour size.

Hattori *et al.* [25] suggested that all tumours with radiological GGA should be classified together as T1a, independent of the whole-tumour size or solid-component size on HRCT, because the prognostic outcome of clinical tumours with GGA was excellent, regardless of these factors. However, their analysis included only a small number of GGA+ tumours with solid portions sized >3 cm.

The current multi-institutional cohort study found that GGA+ tumours with ≤3 cm pathological invasion size had a better prognosis than GGA+ tumours with >3 cm invasion size, so that putting all ‘tumors with GGA’ together would be inappropriate. The prognosis of GGA+ T2a (type 3) tumours was still significantly better than GGA-T2a (type 8) that had the same T factor of type 3, and comparable to that of GGA− T1c (type 7) tumours (5-year OS in ‘All’ cohort: 84.6% vs 83.1%, respectively), as shown in Fig. 3. On the other hand, the prognosis of T2b or larger tumours was unaffected by the absence/presence of GGA. Although the small numbers of patients precluded a definite conclusion regarding the comparison of type 4 versus type 9 tumours in the ‘Eligible’ cohort, the OS of patients with ‘large tumors’ in the ‘All’ cohort was similar to that for NSCLCs with the same T factor, with or without GGA (Fig. 4). The survival impact of GGA within the tumour thus appears to be restricted to tumours with a pathological invasion size ≤4 cm. The prognosis of the tumour with invasive size >4 cm presumably would depend on no longer the presence of GGA but only invasive size (solid component). Further validation studies are necessary to confirm the result in tumours with invasive size >4 cm, especially T2 tumours.

### Limitations

This study had several limitations. First, the data were collected retrospectively from multiple centres, and there may have been interobserver differences in terms of the measurement of pathological invasion size or radiological solid size on HRCT among institutions. Regarding GGA+ tumours, GGA within the tumour on HRCT was not confirmed to correspond to a pathologically non-invasive component of the carcinoma. Second, whole-tumour size ≤2 cm NSCLC was not included in the present study, but the clinicopathological features in NSCLC for whole-tumour size ≤2 cm have been elucidated by many prior studies [5–7, 26]. Third, tumour size was regarded as pathological invasion size in GGA− NSCLC, without actual review of histopathological specimens. Fourth, pleural invasion of PL1 was not reflected in the T descriptor because T category was registered based on the 6th TNM classification in our study. Fifth, detailed information on the GGA status of patients in the JCOG0707 cohort was stored in another database and could not be evaluated. In addition, half of the JCOG0707 cohort received investigational adjuvant chemotherapy with tegafur/gimeracil/oteracil (TS1). However, the OS was similar in the JCOG0707 and ‘Eligible’ cohorts and adjuvant TS1 therapy did not affect the prognosis of patients in the JCOG0707 trial [15], suggesting that these factors were unlikely to result in significant bias. Sixth, since no adjuvant tyrosine kinase inhibitor therapy was recommended at the time of the present study period, EGFR mutation status was not thoroughly investigated. Although our study did not show the survival impact of EGFR mutation per se [15], ADAURA study showed potential OS benefit of adjuvant tyrosine kinase inhibitor in patients with EGFR mutation [27], and future studies would have to incorporate the effect of biology-based postoperative therapies. Seventh, truly ‘real-world, all-comer analysis’ might have to be done including those enrolled in the JCOG0707 trial, but due to the different of data management at different data centres, we could not perform such analyses. This could somewhat undermine the generalizability of our data, by excluding JCOG study-participating subset. Therefore, by showing that the current observational study reproduced the survival outcome of JCOG0707, especially in the ‘Eligible’ cohort, we tried to guarantee that our study population represents real-world patients, both prospective trial-eligible and ineligible population. Finally, since our data were not specifically designed to evaluate the correlation with tumour size and patient outcome according to GGA, they should be taken as hypothesis-generating and not conclusive.

## CONCLUSION

Patients with GGA+ lung cancer with an invasion size ≤4 cm had a better prognosis than those with GGA− solid tumours, for the same T-stage defined by the 8th edition of the TNM classification. However, the presence or absence of GGA within the tumour had little impact on the prognosis in patients with lung cancer with a pathological invasion size or clinical solid size >4 cm. Our data supports that the presence of GGA within the tumour thus only has prognostic significance in patients with an invasion size ≤4 cm. This warrants further investigations, and if validated, should be reflected in future TNM classifications.

## Supplementary Material

ezae158_Supplementary_Data

## Data Availability

The data underlying this article will be shared on reasonable request to the corresponding author.

## ABBREVIATIONS

EGFR: Epidermal growth factor receptor
GGA: Ground-glass area
HR: Hazard ratio
HRCT: High-resolution computed tomography
JCOG: Japan Clinical Oncology Group
NSCLC: Non-small-cell lung cancer
OS: Overall survival
TNM: Tumour, node, metastasis
WHO: World Health Organization

## References

[1] Brierley JD , Gospodarowicz MK, Wittekind C, TNM Classification of Malignant Tumours, 8th edn. New York, NY: Wiley-Blackwell, 2017, 105–12.

[2] Wittekind CH , Compton CC, Brierley JD, Sobin L, TNM supplement. A Commentary on Uniform Use. 4th edn. West Sussex: Wiley-Blackwell, 2012.

[3] Aokage K , Miyoshi T, Ishii G, Kusumoto M, Nomura S, Katsumata S et al Clinical and pathological staging validation in the eighth edition of the TNM classification for lung cancer: correlation between solid size on thin-section computed tomography and invasive size in pathological findings in the new T classification. J Thorac Oncol 2017;12:1403–12. 28627462 10.1016/j.jtho.2017.06.003

[4] Heidinger BH , Anderson KR, Moriarty EM, Costa DB, Gangadharan SP, VanderLaan SP et al Size measurement and T-staging of lung adenocarcinomas manifesting as solid nodules ≤30 mm on CT: radiology-pathology correlation. Acad Radiol 2017;24:851–9. 28256438 10.1016/j.acra.2017.01.009

[5] Suzuki K , Kusumoto M, Watanabe S, Tsuchiya R, Asamura H. Radiologic classification of small adenocarcinoma of the lung: radiologic-pathologic correlation and its prognostic impact. Ann Thorac Surg 2006;81:413–9. 16427823 10.1016/j.athoracsur.2005.07.058

[6] Asamura H , Hishida T, Suzuki K, Koike T, Nakamura K, Kusumoto M et al; Japan Clinical Oncology Group Lung Cancer Surgical Study Group. Radiographically determined noninvasive adenocarcinoma of the lung: survival outcomes of Japan Clinical Oncology Group 0201. J Thorac Cardiovasc Surg 2013;146:24–30. 23398645 10.1016/j.jtcvs.2012.12.047

[7] Sakurai H , Nakagawa K, Watanabe S, Asamura H. Clinicopathologic features of resected subcentimeter lung cancer. Ann Thorac Surg 2015;99:1731–8. 25825199 10.1016/j.athoracsur.2015.01.034

[8] Hattori A , Matsunaga T, Takamochi K, Oh S, Suzuki K. Importance of ground glass opacity component in clinical stage IA radiologic invasive lung cancer. Ann Thorac Surg 2017;104:313–20. 28433219 10.1016/j.athoracsur.2017.01.076

[9] Hattori A , Hirayama S, Matsunaga T, Hayashi T, Takamochi K, Oh S et al Distinct clinicopathologic characteristics and prognosis based on the presence of ground glass opacity component in clinical stage IA lung adenocarcinoma. J Thorac Oncol 2018;14:265–75. 30368010 10.1016/j.jtho.2018.09.026

[10] Yip R , Li K, Liu L, Xu D, Tam K, Yankelevitz D et al Controversies on lung cancers manifesting as part-solid nodules. Eur Radiol 2018;28:747–59. 28835992 10.1007/s00330-017-4975-9 PMC5996385

[11] Ye T , Deng L, Wang S, Xiang J, Zhang Y, Hu H et al Lung adenocarcinomas manifesting as radiological part-solid nodules define a special clinical subtype. J Thorac Oncol 2019;14:617–27. 30659988 10.1016/j.jtho.2018.12.030

[12] Hattori A , Matsunaga T, Fukui M, Takamochi K, Suzuki K. Prognostic influence of a ground-glass opacity component in hypermetabolic lung adenocarcinoma. Eur J Cardiothorac Surg 2022;61:249–56. 34632486 10.1093/ejcts/ezab436

[13] Hishida T , Tsuboi M, Shukuya T, Takamochi K, Sakurai H, Yoh K et al Multicenter observational cohort study of post-operative treatment for completely resected non-small-cell lung cancer of pathological Stage I (T1 >2 cm and T2 in TNM classification version 6). Jpn J Clin Oncol 2015;45:499–501. 25724215 10.1093/jjco/hyv028

[14] Kunitoh H , Tsuboi M, Wakabayashi M, Okada M, Suzuki K, Watanabe S et al; Japan Clinical Oncology Group Lung Cancer Surgical Study Group (JCOG-LCSSG). A phase III study of adjuvant chemotherapy in patients with completely resected, node-negative non-small cell lung cancer (JCOG 0707). JTCVS Open 2020;4:90–102. 36004301 10.1016/j.xjon.2020.08.009 PMC9390442

[15] Shukuya T , Takamochi K, Sakurai H, Yoh K, Hishida T, Tsuboi M et al Efficacy of adjuvant chemotherapy with tegafur-uracil in patients with completely resected, node-negative NSCLC—real-world data in the era of molecularly targeted agents and immunotherapy. JTO Clin Res Rep 2022;3:100320. 35601927 10.1016/j.jtocrr.2022.100320 PMC9117917

[16] Yoh K , Takamochi K, Shukuya T, Hishida T, Tsuboi M, Sakurai H et al Pattern of care in adjuvant therapy for resected Stage I non-small cell lung cancer: real-world data from Japan. Jpn J Clin Oncol 2019;49:63–8. 30452719 10.1093/jjco/hyy165

[17] WHO. World Health Organization classification of tumours. In: Travis WD, Brambilla E (eds). WHO Classification of Tumours of the Lung, Pleura, Thymus and Heart. 4th edn. Lyon: International Agency for Research on Cancer, 2015, 10–151. 10.1097/JTO.0000000000000663 26291007

[18] Asamura H , Nakayama H, Kondo H, Tsuchiya R, Naruke T. Lobe-specific extent of systematic lymph node dissection for non-small cell lung carcinomas according to a retrospective study of metastasis and prognosis. J Thorac Cardiovasc Surg 1999;117:1102–11. 10343258 10.1016/s0022-5223(99)70246-1

[19] Watanabe S , Asamura H. Lymph node dissection for lung cancer: significance, strategy, and technique. J Thorac Oncol 2009;4:652–7. 19357543 10.1097/JTO.0b013e31819cce50

[20] Eba J , Nakamura K. Overview of the ethical guidelines for medical and biological research involving human subjects in Japan. Jpn J Clin Oncol 2022;52:539–44. 35349681 10.1093/jjco/hyac034 PMC9157286

[21] Shuster JJ. Median follow-up in clinical trials. J Clin Oncol 1991;9:191–2. 1985169 10.1200/JCO.1991.9.1.191

[22] Shin KW , Cho S, Chung JH, Lee KW, Lee CT, Kim K et al Comparison of prognosis of solid and part-solid node-negative adenocarcinoma with the same invasive component size. Ann Thorac Surg 2017;103:1654–60. 28131430 10.1016/j.athoracsur.2016.10.040

[23] Ding H , Wang H, Zhang P, Song N, Chen L, Jiang G. Prognostic factors of lung adenocarcinoma manifesting as ground glass nodules larger than 3 cm. Eur J Cardiothorac Surg 2019;55:1130–5. 30561606 10.1093/ejcts/ezy422

[24] Tsurugai Y , Kozuka T, Ishizuka N, Oguchi M. Relationship between the consolidation to maximum tumor diameter ratio and outcomes following stereotactic body radiotherapy for stage I non-small-cell lung cancer. Lung Cancer 2016;92:47–52. 26775596 10.1016/j.lungcan.2015.12.003

[25] Hattori A , Matsunaga T, Takamochi K, Oh S, Suzuki K. Neither maximum tumor size nor solid component size is prognostic in part-solid lung cancer: impact of tumor size should be applied exclusively to solid lung cancer. Ann Thorac Surg 2016;102:407–15. 27177840 10.1016/j.athoracsur.2016.02.074

[26] Saji H , Okada M, Tsuboi M, Nakajima R, Suzuki K, Aokage K et al; West Japan Oncology Group and Japan Clinical Oncology Group. Segmentectomy versus lobectomy in small-sized peripheral non-small-cell lung cancer (JCOG0802/WJOG4607L): a multicentre, open-label, phase 3, randomised, controlled, non-inferiority trial. Lancet 2022;399:1607–17. 35461558 10.1016/S0140-6736(21)02333-3

[27] Tsuboi M , Herbst RS, John T, Kato T, Majem M, Grohé C et al Overall survival with osimertinib in resected EGFR-mutated NSCLC. N Engl J Med 2023;389:137–47. 37272535 10.1056/NEJMoa2304594

