# Supplementary material for: Prognostic significance of ground-glass areas within tumours in non-small-cell lung cancer
Source: Eur J Cardiothorac Surg. 2024 Apr 10;65(4):ezae158. doi: 10.1093/ejcts/ezae158 (PMC11091536; doi:10.1093/ejcts/ezae158)
Supplement: ezae158_Supplementary_Data [file ezae158_supplementary_data.zip › ezae158_Supplementary_Data/Supplementary Table 1.docx]

**Supplementary Table 1. JCOG 0707 eligibility criteria**^14^

Inclusion criteria

1. Pathologically documented NSCLC, except for low-grade malignancy, such as carcinoid tumor, mecoepidermoid carcinoma or adenoid cystic carcinoma.
2. Pathological stage I. The tumor diameter is more than 2cm (T>2cm).
3. Surgical complete resection* is demonstrated by pathological examination of the resected specimen. *: Complete resection is defined as macroscopical and microscopical complete removal of the lung cancer. Cases with microscopically residual tumor or with metastasis at lymph node dissection margin are “incompletely resected”.
4. Tumor was resected with lobectomy, bi-lobectomy or pneumonectomy.
5. Lymph node dissection was performed with ND2a extent or with elective dissection. Elective lymph node dissection removes hilar nodes and selected mediastinal nodes.
6. No prior therapy except for surgical resection.
7. ECOG Performance Status of 0 or 1.
8. Ample organ functions (bone marrow, liver, kidney, lung), as defined below, are confirmed by the latest laboratory tests performed within 14 days of enrollment.
9. Age 20 to 80 years old.
10. Post-operative day 56 or less.
11. Signed informed consent from the patient

Exclusion criteria

1. Concomitant malignancy, defined as synchronous or metachronous malignancy with disease-free period of 5 years or less. Multi-focal lung cancers, including those with Noguchi’s type A or type B, are included in concomitant malignancy. The following are excluded from “concomitant malignancy” category in this protocol: cervical carcinoma in situ judged to be cured by local therapy, gastric or colorectal carcinoma judged to be cured by mucosal resection with endoscopy, non-melanoma skin cancer judged to be cured by local resection.
2. Severe post-operative complication(s), such as infection or stump failure not recovered by time of enrollment.
3. Severe concomitant disease(s), such as interstitial pneumonia, lung fibrosis, intestinal paralysis, ileus, severe diarrhea, uncontrollable diabetes, liver cirrhosis, hepatic failure, renal failure, uncontrollable hypertension, recent (within 6 months) myocardial infarction, unstable angina, cor pulmonale or pulmonary hypertension.
4. Stress electrocardiogram (not specified): positive or could not be performed.
5. Require oxygen, including home oxygen therapy.
6. Receiving systemic corticosteroid of predonisolone 5mg equivalent or more.
7. Past history of grade 3 or more drug allergy.
8. Continuous flucytosine, phenytoin or warfarin potassium treatment.
9. Pregnant/possibly pregnant woman, breast-feeding woman, woman with intention for pregnancy, man with intention for making pregnant.
10. Psychiatric disease or psychiatric symptoms prohibiting participation to the trial.
11. Positive for human immunodeficiency virus (documentation of negativity by serum test is not mandatory).
12. Positive for hepatitis virus B surface antigen.
