# Supplementary material for: Prognostic significance of ground-glass areas within tumours in non-small-cell lung cancer
Source: Eur J Cardiothorac Surg. 2024 Apr 10;65(4):ezae158. doi: 10.1093/ejcts/ezae158 (PMC11091536; doi:10.1093/ejcts/ezae158)
Supplement: ezae158_Supplementary_Data [file ezae158_supplementary_data.zip › ezae158_Supplementary_Data/Supplementary Table 2.docx]

**Supplementary Table 2. Classification of Lung Cancers According to High-Resolution Computed Tomography Type**

| Type | Number | Whole-tumor size | Intratumoral GGA on HRCT | Pathological invasion size | T factor  (8^th^ TNM) |
| --- | --- | --- | --- | --- | --- |
| 1 | 1309 | >2 cm, ≤ 3 cm | Present | ≤ 3 cm | T1a-c |
| 2 | 470 | > 3 cm | Present | ≤ 3 cm | T1a-c |
| 3 | 138 | > 3cm | Present | > 3 cm, ≤ 4 cm | T2a |
| 4 | 42 | > 3 cm | Present | > 4 cm, ≤ 5 cm | T2b |
| 5 | 25 | > 3 cm | Present | > 5cm, ≤ 7 cm | T3 |
| 6 | 13 | > 3 cm | Present | > 7 cm | T4 |
| 7 | 1420 | >2 cm, ≤ 3 cm | Absent | >2 cm, ≤ 3 cm | T1c |
| 8 | 817 | > 3 cm, ≤ 4 cm | Absent | > 3 cm, ≤ 4 cm | T2a |
| 9 | 367 | > 4 cm, ≤ 5 cm | Absent | > 4 cm, ≤ 5 cm | T2b |
| 10 | 188 | > 5 cm, ≤ 7 cm | Absent | > 5 cm, ≤ 7 cm | T3 |
| 11 | 65 | > 7 cm | Absent | > 7 cm | T4 |

HRCT, high-resolution computed tomography; GGA, ground-glass area; TNM, tumor, node, metastasis.
