# Supplementary material for: Prognostic significance of ground-glass areas within tumours in non-small-cell lung cancer
Source: Eur J Cardiothorac Surg. 2024 Apr 10;65(4):ezae158. doi: 10.1093/ejcts/ezae158 (PMC11091536; doi:10.1093/ejcts/ezae158)
Supplement: ezae158_Supplementary_Data [file ezae158_supplementary_data.zip › ezae158_Supplementary_Data/Supplmentary FIG1.pdf]

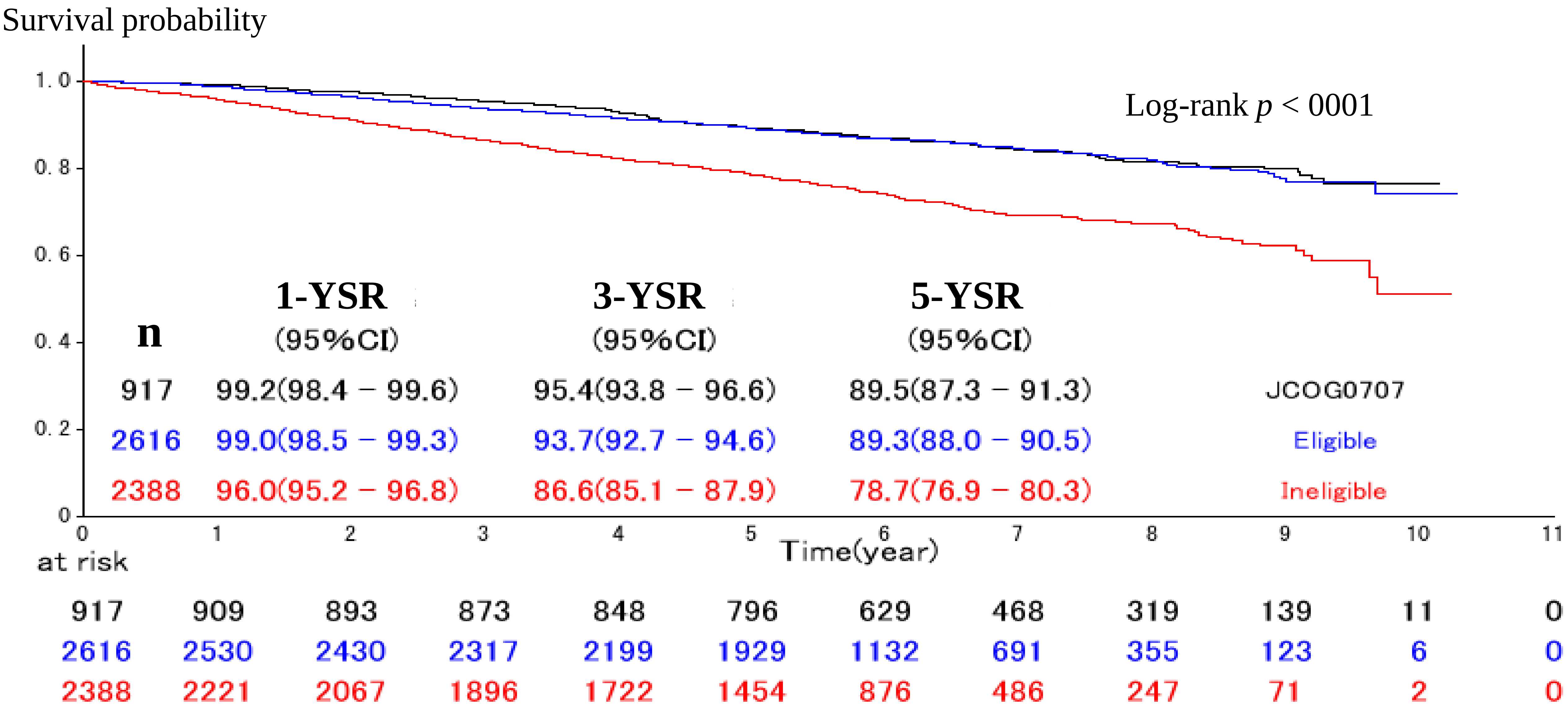

Supplementary Figure 1. Overall survival curves for “Eligible”, “Ineligible”, and “JCOG0707” cohorts. The 5-year survival rates of the “Eligible”, “Ineligible”, and “JCOG0707” cohorts were 89.4%, 78.7%, and 89.5%, respectively. The “Ineligible” cohort had a significantly poorer prognosis than the “Eligible” and “JCOG0707” cohorts. YSR, year survival rate; n, number; CI, confidence interval; JCOG, Japan Clinical Oncology Group.
