# Supplementary material for: Prognostic significance of ground-glass areas within tumours in non-small-cell lung cancer
Source: Eur J Cardiothorac Surg. 2024 Apr 10;65(4):ezae158. doi: 10.1093/ejcts/ezae158 (PMC11091536; doi:10.1093/ejcts/ezae158)
Supplement: ezae158_Supplementary_Data [file ezae158_supplementary_data.zip › ezae158_Supplementary_Data/Supplmentary FIG2.pdf]

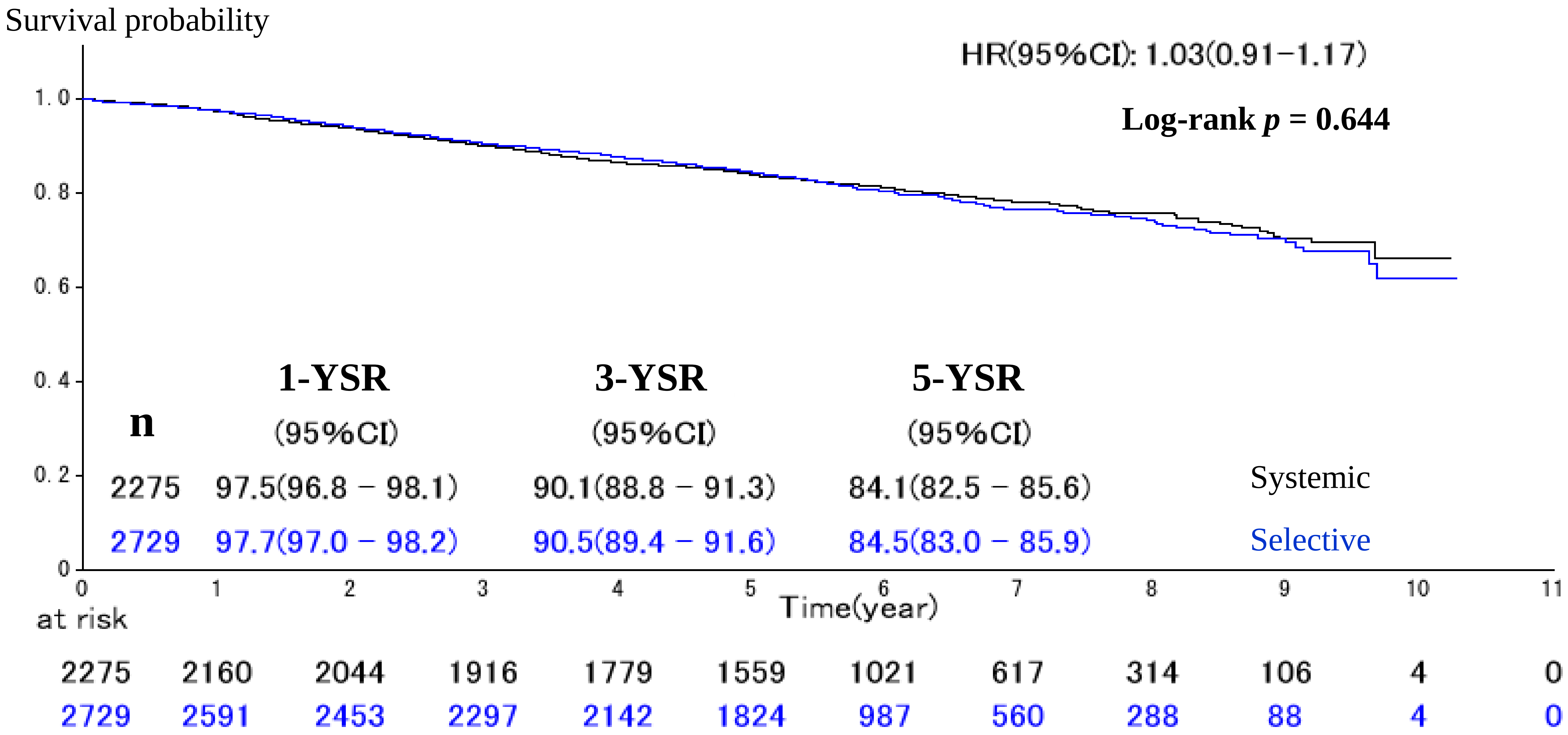

Supplementary Figure 2. Overall survival curves according to mode of lymph node dissection. The 5-year survival rates were 84.1% for systematic lymph node dissection and 84.5% for selective lymph node dissection. The difference in survival was not significant (p = 0.644). HR, hazard ratio; CI, confidence interval; YSR, year survival rate; n, number.
